# Supplementary material for: Metabolomic Profile of BALB/c Macrophages Infected with Leishmania amazonensis: Deciphering L-Arginine Metabolism
Source: Int J Mol Sci. 2019 Dec 11;20(24):6248. doi: 10.3390/ijms20246248 (PMC6940984; doi:10.3390/ijms20246248)
Supplement: Supplementary file 1 [file ijms-20-06248-s001.pdf]

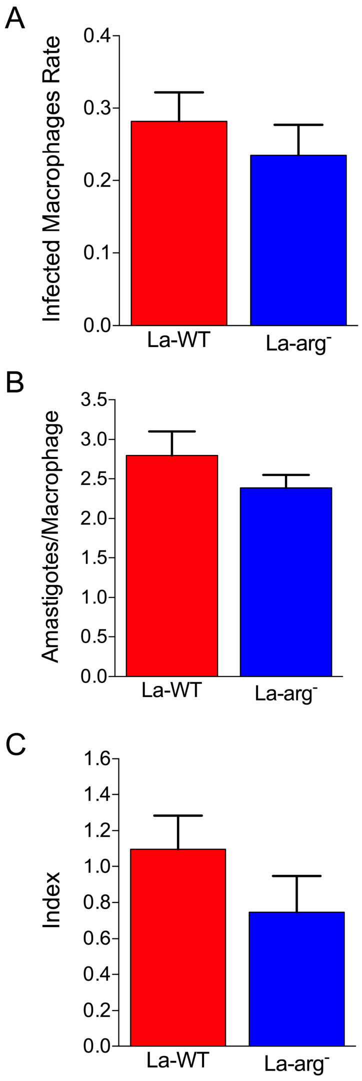

Supplementary Figure 1. Infectivity of BALB/c-BMDMs infected with *La*-WT and *La-arg*⁻ *L. amazonensis*. BMDMs (2 × 10^5) were plated into chamber slides overnight and infected with *La*-WT or *La-arg*⁻ *L. amazonensis* (MOI 5:1). After 4 h, the cultures were washed, fixed and stained with Giemsa, and the index of infection was determined via microscopic counting of infected macrophages and amastigotes per macrophage. (**A**) Percentage of infected macrophages. (**B**) Number of amastigotes per infected macrophages. (**C**) Infectivity index. The data represents the mean ± SEM of 6 replicates from 3 independent experiments (*n* = 1000 macrophages/replicate).

A

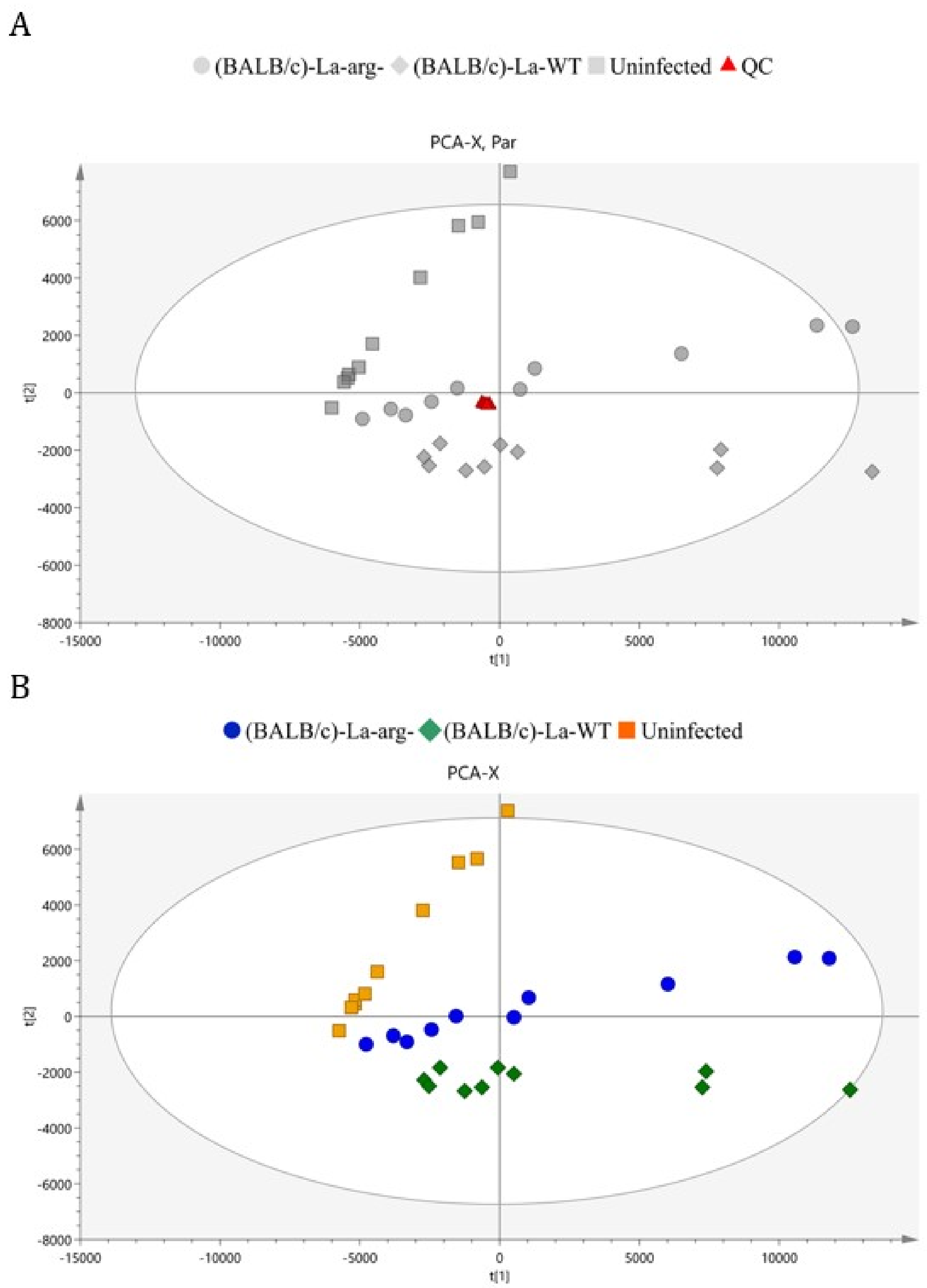

Supplementary Figure 2. (**A**) PCA-X score map of *L. amazonensis* infected macrophage and quality control samples (QC, triangle) using pareto scaling in non-normalized setting (**B**) PCA-X score plot of *L. amazonensis* wild type ((BALB/c)- La-WT-infected, green diamond), arginase knockout ((BALB/c)-La-arg^{-}-infected, blue circle), uninfected BALB/c macrophages (Uninfected, orange squares).

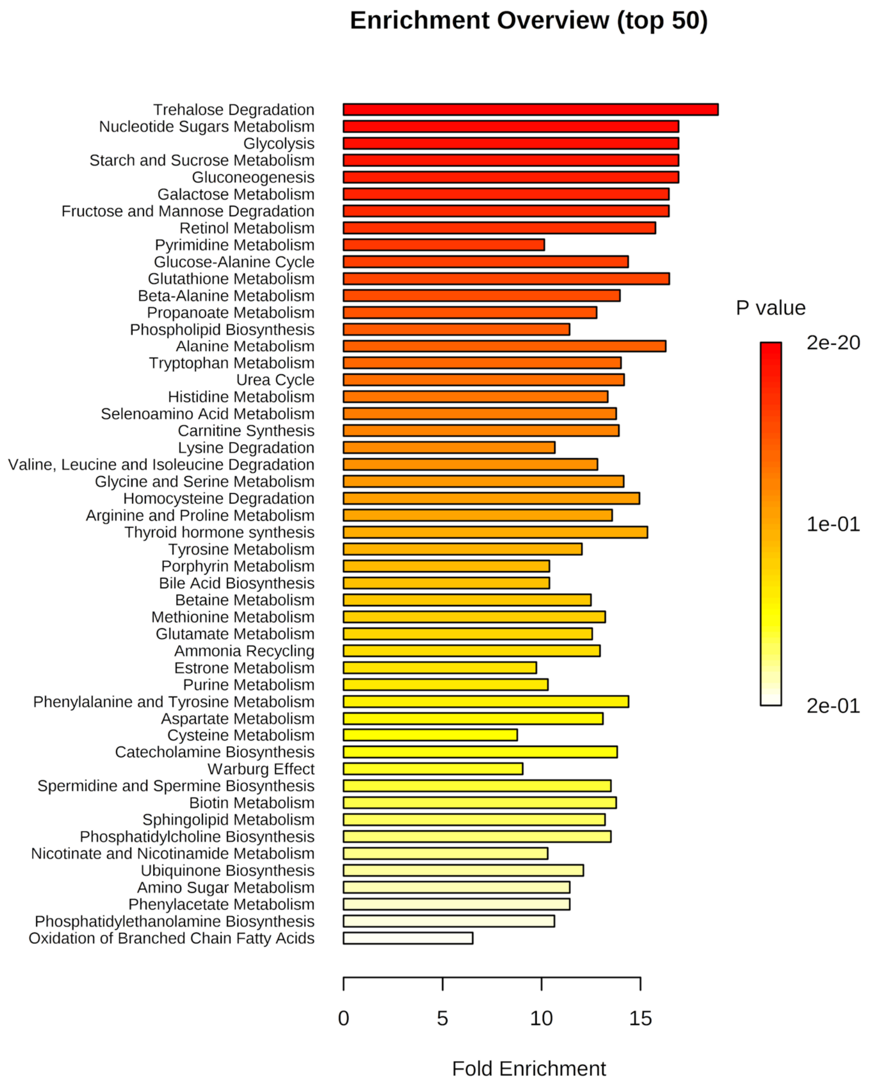

Supplementary Figure 3. Pathway associated metabolite enrichment in *L. amazonensis* infected and uninfected macrophages. Enrichment analysis of dysregulated pathways based in metabolite peak areas from *L. amazonensis* wild type ((BALB/c)-La-WT-infected) and uninfected BALB/c (uninfected) macrophages using a continuous regression in pathway-associated metabolite sets in MetaboloAnalyst 4.0 software (http://www.metaboanalyst.ca/faces/Secure/time/Heatmap2View.xhtml).

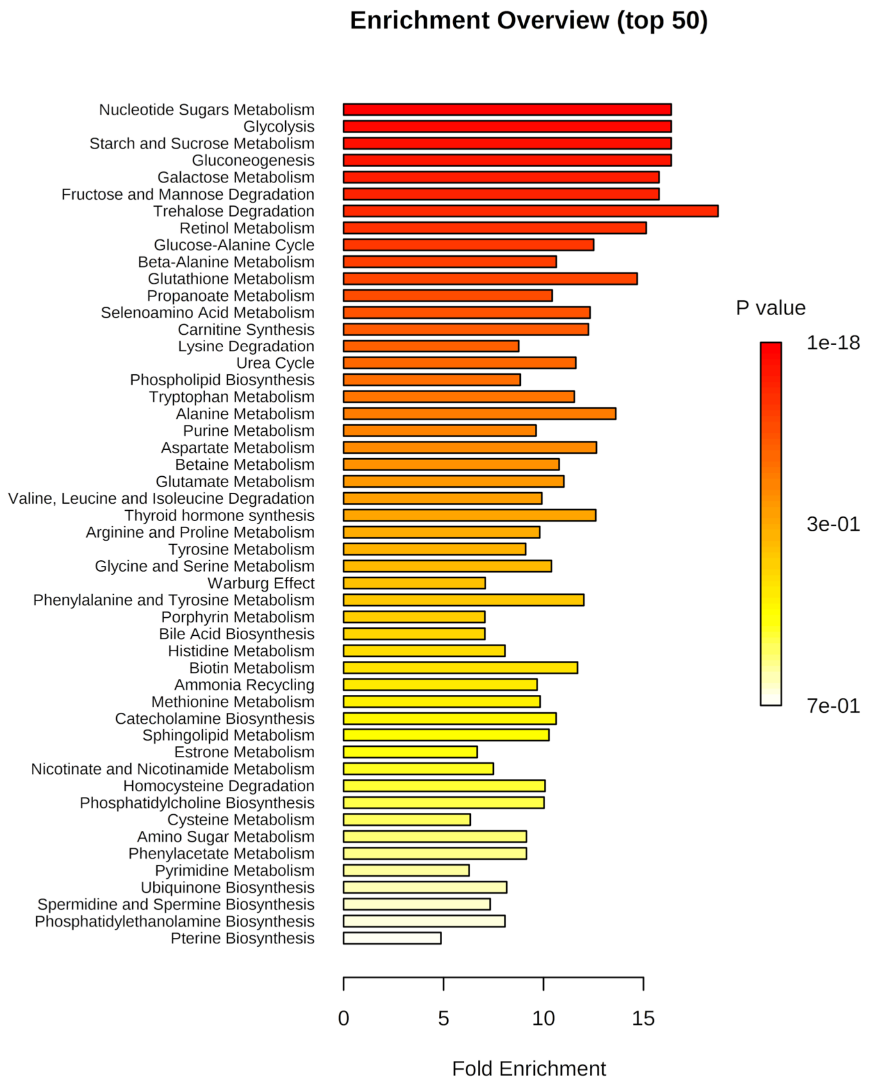

Supplementary Figure 4. Pathway associated metabolite enrichment in *L. amazonensis* infected and uninfected macrophages. Enrichment analysis of dysregulated pathways based in metabolite peak areas from *L. amazonensis* arginase knockout ((BALB/c)-La-arg^{-}-infected) and uninfected BALB/c (uninfected) macrophages using a continuous regression in pathway-associated metabolite sets in MetaboloAnalyst 4.0 software (http://www.metaboanalyst.ca/faces/Secure/time/Heatmap2View.xhtml).

Suplemmetary Table 1: Associated-Pathway of Quantitative Enrichment Analysis from significantly regulated metabolites during *L. amazonensis* infection. Enrichment Analysis of dysregulated pathways based in metabolites peak areas from *L. amazonensis* wild type ((BALB/c)-La-WT-infected) and arginase knockout ((BALB/c)-La-arg^{-}-infected) using a continuous regression in pathway-associated metabolite sets in MetaboloAnalyst 4.0 software (http://www.metaboanalyst.ca/faces/Secure/time/Heatmap2View.xhtml).

	Total Cmpd	Hits	Statistic Q	Expected Q	Raw p	Holm p	FDR
**Arginine and Proline Metabolism**	53	7	32.84	5.26	6.71×10^{-4}	5.24×10^{-2}	5.24×10^{-2}
**Urea Cycle**	29	6	29.88	5.26	1.63×10^{-3}	1.25×10^{-2}	6.34×10^{-2}
**Histidine Metabolism**	43	4	35.16	5.26	2.95×10^{-3}	2.24×10^{-2}	6.92×10^{-2}
**Beta-Alanine Metabolism**	34	2	32.55	5.26	4.39×10^{-3}	3.29×10^{-3}	6.92×10^{-2}
**Spermidine and Spermine Biosynthesis**	18	5	32.91	5.26	4.43×10^{-3}	3.29×10^{-3}	6.92×10^{-2}
**Galactose Metabolism**	38	3	21.67	5.26	1.57×10^{-2}	1	1.75×10^{-2}
**Fructose and Mannose Degradation**	32	3	21.67	5.26	1.57×10^{-2}	1	1.75×10^{-2}

**Glycine and Serine Metabolism**	59	10	21.98	5.26	1.85×10^{-2}	1	1.80×10^{-2}
**Aspartate Metabolism**	35	4	18.19	5.26	4.03×10^{-2}	1	3.49×10^{-2}
**Trehalose Degradation**	11	1	17.10	5.26	6.99×10^{-2}	1	4.97×10^{-2}
**Metabolism Metabolism**	21	4	16.19	5.26	7.00×10^{-2}	1	4.97×10^{-2}
**Ammonia Recycling**	32	6	14.58	5.26	8.40×10^{-2}	1	5.10×10^{-2}
**Homocysteine Degradation**	9	2	13.78	5.26	1.01×10^{-1}	1	5.10×10^{-2}
**Nucleotide Sugars Metabolism**	20	2	12.10	5.26	1.11×10^{-1}	1	5.10×10^{-2}
**Glycolysis**	25	2	12.10	5.26	1.11×10^{-1}	1	5.10×10^{-2}
**Starch and Sucrose Metabolism**	31	2	12.10	5.26	1.11×10^{-1}	1	5.10×10^{-2}
**Gluconeogenesis Metabolism**	35	2	12.10	5.26	1.11×10^{-1}	1	5.10×10^{-2}
**Glutamate Metabolism**	49	6	10.80	5.26	1.39×10^{-1}	1	6.03×10^{-2}
**Methionine**	43	10	9.92	5.26	1.62×10^{-1}	1	6.47×10^{-2}
**Glucose-Alanine Cycle**	13	2	9.76	5.26	1.70×10^{-1}	1	6.47×10^{-2}
**Pyrimidine Metabolism**	59	2	9.37	5.26	1.77×10^{-1}	1	6.47×10^{-2}
**Tryptophan Metabolism**	60	4	9.34	5.26	1.83×10^{-1}	1	6.47×10^{-2}
**Ubiquinone Biosynthesis**	20	1	9.29	5.26	1.91×10^{-1}	1	6.48×10^{-2}
**Alanine Metabolism**	17	2	8.31	5.26	2.17×10^{-1}	1	7.04×10^{-2}
**Valine, Leucine and Isoleucine Degradation**	60	3	7.56	5.26	2.35×10^{-1}	1	7.32×10^{-2}
**Betaine Metabolism**	21	5	6.93	5.26	2.52×10^{-1}	1	7.55×10^{-2}
**Retinol Metabolism**	37	3	6.44	5.26	2.75×10^{-1}	1	7.68×10^{-2}
**Selenoamino Acid Metabolism**	28	4	6.45	5.26	2.76×10^{-1}	1	7.68×10^{-2}
**Estrone Metabolism**	24	2	6.04	5.26	3.04×10^{-1}	1	8.06×10^{-2}
**Oxidation of Branched Chain Fatty Acids**	26	1	5.66	5.26	3.12×10^{-1}	1	8.06×10^{-2}
**Cysteine Metabolism**	26	2	5.77	5.26	3.22×10^{-1}	1	8.06×10^{-2}
**Catecholamine Biosynthesis**	20	2	5.26	5.26	3.31×10^{-1}	1	8.06×10^{-2}
**Nicotinate and Nicotinamide Metabolism**	37	3	5.05	5.26	3.51×10^{-1}	1	8.28×10^{-2}
**Tyrosine Metabolism**	72	3	4.39	5.26	3.87×10^{-1}	1	8.64×10^{-1}
**Amino Sugar Metabolism**	33	1	3.99	5.26	3.99×10^{-1}	1	8.64×10^{-1}
**Phenylacetate Metabolism**	9	1	3.99	5.26	3.99×10^{-1}	1	8.64×10^{-1}
**Purine Metabolism**	74	6	3.99	5.26	4.15×10^{-1}	1	8.74×10^{-1}
**Phosphatidylcholine Biosynthesis**	14	2	3.19	5.26	4.57×10^{-1}	1	9.31×10^{-1}
**Thyroid hormone synthesis**	13	1	2.89	5.26	4.74×10^{-1}	1	9.31×10^{-1}
**Carnitine Synthesis**	22	5	2.99	5.26	4.83×10^{-1}	1	9.31×10^{-1}
**Phenylalanine and Tyrosine Metabolism**	28	2	2.69	5.26	4.89×10^{-1}	1	9.31×10^{-1}
**Phosphatidylethanolamine Biosynthesis**	12	1	2.21	5.26	5.31×10^{-1}	1	9.68×10^{-1}
**Warburg Effect**	58	2	2.89	5.26	5.49×10^{-1}	1	9.68×10^{-1}
**Porphyrin Metabolism**	40	2	2.52	5.26	5.58×10^{-1}	1	9.68×10^{-1}
**Bile Acid Biosynthesis**	65	2	2.52	5.26	5.58×10^{-1}	1	9.68×10^{-1}
**Sphingolipid Metabolism**	40	2	1.25	5.26	6.57×10^{-1}	1	9.84×10^{-1}
**Biotin Metabolism**	8	1	1.09	5.26	6.61×10^{-1}	1	9.84×10^{-1}
**Propanoate Metabolism**	42	2	1.17	5.26	7.22×10^{-1}	1	9.84×10^{-1}
**Lysine Degradation**	30	2	0.87	5.26	8.04×10^{-1}	1	9.84×10^{-1}
**Phospholipid Biosynthesis**	29	2	0.48	5.26	8.70×10^{-1}	1	9.84×10^{-1}
**Pterine Biosynthesis**	29	1	0.00	5.26	9.84×10^{-1}	1	9.84×10^{-1}
**Inositol Metabolism**	33	1	0.00	5.26	9.84×10^{-1}	1	9.84×10^{-1}
**Caseine Metabolism**	24	1	0.00	5.26	9.84×10^{-1}	1	9.84×10^{-1}
**Glycerolipid Metabolism**	25	1	0.00	5.26	9.84×10^{-1}	1	9.84×10^{-1}
**Fatty acid Metabolism**	43	1	0.00	5.26	9.84×10^{-1}	1	9.84×10^{-1}
**Folate Metabolism**	29	1	0.00	5.26	9.84×10^{-1}	1	9.84×10^{-1}
**Fatty Acid Elongation in Mitrochrondria**	35	1	0.00	5.26	9.84×10^{-1}	1	9.84×10^{-1}
**Citric Acid Cycle**	32	1	0.00	5.26	9.84×10^{-1}	1	9.84×10^{-1}
**Pyruvate Metabolism**	48	1	0.00	5.26	9.84×10^{-1}	1	9.84×10^{-1}
**Androgen and Estrogen Metabolism**	33	1	0.00	5.26	9.84×10^{-1}	1	9.84×10^{-1}
**Ketone Body Metabolism**	13	1	0.00	5.26	9.84×10^{-1}	1	9.84×10^{-1}
**Butyrate Metabolism**	19	1	0.00	5.26	9.84×10^{-1}	1	9.84×10^{-1}
**Glycerol Phosphate Shuttle**	11	1	0.00	5.26	9.84×10^{-1}	1	9.84×10^{-1}
**Malate-Aspartate Shuttle**	10	1	0.00	5.26	9.84×10^{-1}	1	9.84×10^{-1}
**Steroidogenesis**	43	1	0.00	5.26	9.84×10^{-1}	1	9.84×10^{-1}
**Mitochondrial Electron Transport Chain**	19	1	0.00	5.26	9.84×10^{-1}	1	9.84×10^{-1}
**Ethanol Degradation**	19	1	0.00	5.26	9.84×10^{-1}	1	9.84×10^{-1}
**Phytanic Acid Peroxisomal Oxidation**	26	1	0.00	5.26	9.84×10^{-1}	1	9.84×10^{-1}
**Threonine and 2-Oxobutanoate Degradation**	20	1	0.00	5.26	9.84×10^{-1}	1	9.84×10^{-1}
**Inositol Phosphate Metabolism**	26	1	0.00	5.26	9.84×10^{-1}	1	9.84×10^{-1}
**Transfer of Acetyl Groups into Mitochondria**	22	1	0.00	5.26	9.84×10^{-1}	1	9.84×10^{-1}
**Plasmalogen Synthesis**	26	1	0.00	5.26	9.84×10^{-1}	1	9.84×10^{-1}
**Mitochondrial Beta-Oxidation of Short Chain Saturated Fatty Acids**	27	1	0.00	5.26	9.84×10^{-1}	1	9.84×10^{-1}
**Mitochondrial Beta-Oxidation of Medium Chain Saturated Fatty Acids**	27	1	0.00	5.26	9.84×10^{-1}	1	9.84×10^{-1}

**Mitochondrial Beta-Oxidation of Long Chain Saturated Fatty Acids**	28	1	0.00	5.26	9.84×10^{-1}	1	9.84×10^{-1}
**De Novo Triacylglycerol Biosynthesis**	9	1	0.00	5.26	9.84×10^{-1}	1	9.84×10^{-1}
**Cardiolipin Biosynthesis**	11	1	0.00	5.26	9.84×10^{-1}	1	9.84×10^{-1}
**Androstenedione Metabolism**	24	1	0.00	5.26	9.84×10^{-1}	1	9.84×10^{-1}

Suplemmetary Table 2: Associated-Pathway of Quantitative Enrichment Analysis from significantly regulated metabolites during *L. amazonensis* infection. Enrichment Analysis of dysregulated pathways based in metabolites peak areas from *L. amazonensis* wild type ((BALB/c)-La-WT-infected) and uninfected BALB/c (Uninfected) macrophages using a continuous regression in pathway-associated metabolite sets in MetaboloAnalyst 4.0 software (http://www.metaboanalyst.ca/faces/Secure/time/Heatmap2View.xhtml).

	**Total Cmpd**	**Hits**	**Statistic Q**	**Expected Q**	**Raw p**	**Holm p**	**FDR**
**Trehalose Degradation**	11	1	99.52	5.26	2.79×10^{-22}	2.17×10^{-20}	2.17×10^{-20}
**Nucleotide Sugars Metabolism**	20	2	89.07	5.26	4.99×10^{-19}	3.84×10^{-17}	7.78×10^{-18}
**Glycolysis**	25	2	89.07	5.26	4.99×10^{-19}	3.84×10^{-17}	7.78×10^{-18}
**Starch and Sucrose Metabolism**	31	2	89.07	5.26	4.99×10^{-19}	3.84×10^{-17}	7.78×10^{-18}
**Gluconeogenesis**	35	2	89.07	5.26	4.99×10^{-19}	3.84×10^{-17}	7.78×10^{-18}
**Galactose Metabolism**	38	3	86.47	5.26	1.10×10^{-17}	7.78×10^{-16}	7.78×10^{-16}
**Fructose and Mannose Degradation**	32	3	86.47	5.26	1.10×10^{-17}	7.78×10^{-18}	1.22×10^{-16}
**Retinol Metabolism**	37	3	82.91	5.26	2.93×10^{-17}	2.08×10^{-15}	2.85×10^{-16}
**Pyrimidine Metabolism**	59	2	53.34	5.26	1.87×10^{-12}	1.31×10^{-10}	1.48×10^{-11}
**Glucose-Alanine Cycle**	13	2	75.66	5.26	1.90×10^{-12}	1.31×10^{-10}	1.48×10^{-11}
**Glutathione Metabolism**	21	4	86.61	5.26	1.30×10^{-11}	8.82×10^{-10}	9.19×10^{-11}
**Beta-Alanine Metabolism**	34	2	73.46	5.26	4.34×10^{-11}	2.91×10^{-9}	2.82×10^{-10}
**Propanoate Metabolism**	42	2	67.28	5.26	4.71×10^{-10}	3.11×10^{-8}	2.82×10^{-9}
**Phospholipid Biosynthesis**	29	2	60.05	5.26	9.81×10^{-10}	6.38×10^{-8}	5.47×10^{-9}
**Alanine Metabolism**	17	2	85.66	5.26	1.64×10^{-9}	1.05×10^{-7}	8.51×10^{-9}
**Tryptophan Metabolism**	60	4	73.72	5.26	2.91×10^{-9}	1.83×10^{-7}	1.42×10^{-8}
**Urea Cycle**	29	6	74.57	5.26	3.84×10^{-9}	2.38×10^{-7}	1.76×10^{-8}
**Histidine Metabolism**	43	4	70.20	5.26	4.77×10^{-9}	2.91×10^{-7}	2.03×10^{-8}
**Selenoamino Acid Metabolism**	28	4	72.46	5.26	4.95×10^{-9}	2.97×10^{-7}	2.03×10^{-8}
**Carnitine Synthesis**	22	5	73.18	5.26	6.55×10^{-9}	3.86×10^{-7}	2.55×10^{-8}
**Lysine Degradation**	30	2	56.17	5.26	3.85×10^{-8}	2.23×10^{-6}	1.38×10^{-7}
**Valine, Leucine and Isoleucine Degradation**	60	3	67.49	5.26	3.90×10^{-8}	2.23×10^{-6}	1.38×10^{-7}
**Glycine and Serine Metabolism**	59	10	74.46	5.26	4.33×10^{-8}	2.42×10^{-6}	1.42×10^{-7}
**Homocysteine Degradation**	9	2	78.68	5.26	4.37×10^{-8}	2.42×10^{-6}	1.42×10^{-7}
**Arginine and Proline Metabolism**	53	7	71.39	5.26	4.92×10^{-8}	2.66×10^{-6}	1.53×10^{-7}
**Thyroid hormone synthesis**	13	1	80.79	5.26	7.28×10^{-8}	3.86×10^{-6}	2.18×10^{-7}
**Tyrosine Metabolism**	72	3	63.36	5.26	7.71×10^{-8}	4.01×10^{-6}	2.23×10^{-7}
**Porphyrin Metabolism**	40	2	54.68	5.26	1.32×10^{-7}	6.71×10^{-6}	3.54×10^{-7}
**Bile Acid Biosynthesis**	65	2	54.68	5.26	1.32×10^{-7}	6.71×10^{-6}	3.54×10^{-7}
**Betaine Metabolism**	21	5	65.78	5.26	1.71×10^{-7}	8.37×10^{-6}	4.44×10^{-7}
**Methionine Metabolism**	43	10	69.56	5.26	2.08×10^{-7}	9.96×10^{-6}	5.22×10^{-7}
**Glutamate Metabolism**	49	6	66.11	5.26	2.49×10^{-7}	1.17×10^{-5}	5.91×10^{-7}
**Ammonia Recycling**	32	6	68.14	5.26	2.50×10^{-7}	1.17×10^{-5}	5.91×10^{-7}
**Estrone Metabolism**	24	2	51.25	5.26	2.97×10^{-7}	1.34×10^{-5}	6.81×10^{-7}
**Purine Metabolism**	74	6	54.32	5.26	5.08×10^{-7}	2.24×10^{-5}	1.11×10^{-6}
**Phenylalanine and Tyrosine Metabolism**	28	2	75.73	5.26	5.14×10^{-7}	2.24×10^{-5}	1.11×10^{-6}
**Aspartate Metabolism**	35	4	68.94	5.26	7.14×10^{-7}	3.00×10^{-5}	1.50×10^{-6}
**Cysteine Metabolism**	26	2	46.15	5.26	8.57×10^{-7}	3.51×10^{-5}	1.76×10^{-6}
**Catecholamine Metabolism**	20	2	72.74	5.26	1.01×10^{-6}	4.06×10^{-5}	2.03×10^{-6}
**Warburg Effect**	58	2	47.60	5.26	1.18×10^{-6}	4.60×10^{-5}	2.30×10^{-6}
**Spermidine and Spermine Biosynthesis**	18	5	71.06	5.26	1.38×10^{-6}	5.26×10^{-5}	2.63×10^{-6}
**Biotin Metabolism**	8	1	72.47	5.26	1.95×10^{-6}	7.20×10^{-5}	3.62×10^{-6}
**Sphingolipid Metabolism**	40	2	69.48	5.26	2.01×10^{-6}	7.23×10^{-5}	3.64×10^{-6}
**Phosphatidylcholine Biosynthesis**	14	2	71.08	5.26	2.41×10^{-6}	8.42×10^{-5}	4.26×10^{-6}
**Nicotinate and Nicotinamide Metabolism**	37	3	54.24	5.26	2.77×10^{-6}	9.41×10^{-5}	4.80×10^{-6}
**Ubiquinone Biosynthesis**	20	1	63.73	5.26	2.45×10^{-5}	8.10×10^{-4}	4.16×10^{-5}
**Amino Sugar Metabolism**	33	1	60.12	5.26	5.91×10^{-5}	1.89×10^{-3}	9.61×10^{-5}
**Phenylacetate Metabolism**	9	1	60.12	5.26	5.91×10^{-5}	1.89×10^{-4}	9.61×10^{-5}
**Phosphatidylethanolamine Biosynthesis**	12	1	56.04	5.26	1.46×10^{-4}	4.39×10^{-3}	2.33×10^{-4}
**Oxidation of Branched Chain Fatty Acids**	26	1	34.31	5.26	6.65×10^{-3}	1.93×10^{-1}	1.04×10^{-2}
**Pterine Biosynthesis**	29	1	30.76	5.26	1.12×10^{-2}	3.12×10^{-1}	1.12×10^{-2}
**Inositol Metabolism**	33	1	30.76	5.26	1.12×10^{-2}	3.12×10^{-1}	1.12×10^{-2}
**Caseine Metabolism**	24	1	30.76	5.26	1.12×10^{-2}	3.12×10^{-1}	1.12×10^{-2}
**Glycerolipid Metabolism**	25	1	30.76	5.26	1.12×10^{-2}	3.12×10^{-1}	1.12×10^{-2}
**Fatty acid Metabolism**	43	1	30.76	5.26	1.12×10^{-2}	3.12×10^{-1}	1.12×10^{-2}
**Folate Metabolism**	29	1	30.76	5.26	1.12×10^{-2}	3.12×10^{-1}	1.12×10^{-2}

**Fatty Acid Elongation In Mitochondria**	35	1	30.76	5.26	1.12×10^{-2}	3.12×10^{-1}	1.12×10^{-2}
**Citric Acid Cycle**	32	1	30.76	5.26	1.12×10^{-2}	3.12×10^{-1}	1.12×10^{-2}
**Pyruvate Metabolism**	48	1	30.76	5.26	1.12×10^{-2}	3.12×10^{-1}	1.12×10^{-2}
**Androgen and Estrogen Metabolism**	33	1	30.76	5.26	1.12×10^{-2}	3.12×10^{-1}	1.12×10^{-2}
**Ketone Body Metabolism**	13	1	30.76	5.26	1.12×10^{-2}	3.12×10^{-1}	1.12×10^{-2}
**Butyrate Metabolism**	19	1	30.76	5.26	1.12×10^{-2}	3.12×10^{-1}	1.12×10^{-2}
**Glycerol Phosphate Shuttle**	11	1	30.76	5.26	1.12×10^{-2}	3.12×10^{-1}	1.12×10^{-2}
**Malate-Aspartate Shuttle**	10	1	30.76	5.26	1.12×10^{-2}	3.12×10^{-1}	1.12×10^{-2}
**Steroidogenesis**	43	1	30.76	5.26	1.12×10^{-2}	3.12×10^{-1}	1.12×10^{-2}
**Mitochondrial Electron Transport Chain**	19	1	30.76	5.26	1.12×10^{-2}	3.12×10^{-1}	1.12×10^{-2}
**Ethanol Degradation**	19	1	30.76	5.26	1.12×10^{-2}	3.12×10^{-1}	1.12×10^{-2}
**Phytanic Acid Peroxisomal Oxidation**	26	1	30.76	5.26	1.12×10^{-2}	3.12×10^{-1}	1.12×10^{-2}
**Threonine and 2-Oxobutanoate Degradation**	20	1	30.76	5.26	1.12×10^{-2}	3.12×10^{-1}	1.12×10^{-2}
**Inositol Phosphate Metabolism**	26	1	30.76	5.26	1.12×10^{-2}	3.12×10^{-1}	1.12×10^{-2}
**Transfer of Acetyl Groups into Mitochondria**	22	1	30.76	5.26	1.12×10^{-2}	3.12×10^{-1}	1.12×10^{-2}
**Plasmalogen Synthesis**	26	1	30.76	5.26	1.12×10^{-2}	3.12×10^{-1}	1.12×10^{-2}
**Mitochondrial Beta-Oxidation of Short Chain Saturated Fatty Acids**	27	1	30.76	5.26	1.12×10^{-2}	3.12×10^{-1}	1.12×10^{-2}
**Mitochondrial Beta-Oxidation of Medium Chain Saturated Fatty Acids**	27	1	30.76	5.26	1.12×10^{-2}	3.12×10^{-1}	1.12×10^{-2}
**Mitochondrial Beta-Oxidation of Long Chain Saturated Fatty Acids**	28	1	30.76	5.26	1.12×10^{-2}	3.12×10^{-1}	1.12×10^{-2}
**De Novo Triacylglycerol Biosynthesis**	9	1	30.76	5.26	1.12×10^{-2}	3.12×10^{-1}	1.12×10^{-2}
**Cardiolipin Biosynthesis**	11	1	30.76	5.26	1.12×10^{-2}	3.12×10^{-1}	1.12×10^{-2}
**Androstenedione Metabolism**	24	1	30.76	5.26	1.12×10^{-2}	3.12×10^{-1}	1.12×10^{-2}

Suplemmetary Table 3: Associated-Pathway of Quantitative Enrichment Analysis from significantly regulated metabolites during *L. amazonensis* infection. Enrichment Analysis of dysregulated pathways based in metabolites peak areas from *L. amazonensis* arginase knockout ((BALB/c)-La-arg$^-$-infected) and uninfected BALB/c (Uninfected) macrophages using a continuous regression in pathway-associated metabolite sets in MetaboloAnalyst 4.0 software (http://www.metaboanalyst.ca/faces/Secure/time/Heatmap2View.xhtml).

	**Total Cmpd**	**Hits**	**Statistic Q**	**Expected Q**	**Rawp**	**Holm p**	**FDR**
**Nucleotide Sugars Metabolism**	20	2	86.25	5.26	1.28×10^{-20}	9.96×10^{-19}	2.49×10^{-19}
**Glycolysis**	25	2	86.25	5.26	1.28×10^{-20}	9.96×10^{-19}	2.49×10^{-19}
**Starch and Sucrose Metabolism**	31	2	86.25	5.26	1.28×10^{-20}	9.96×10^{-19}	2.49×10^{-19}
**Gluconeogenesis**	35	2	86.25	5.26	1.28×10^{-20}	9.96×10^{-19}	2.49×10^{-19}
**Galactose Metabolism**	38	3	83.04	5.26	5.40×10^{-20}	3.99×10^{-18}	$7.02\ 10^{-19}$
**Fructose and Mannose Degradation**	32	3	83.04	5.26	5.40×10^{-20}	3.99×10^{-18}	$7.02\ 10^{-19}$
**Trehalose Degradation**	11	1	98.56	5.26	5.08×10^{-18}	3.66×10^{-16}	5.66×10^{-17}
**Retinol Metabolism**	37	3	79.65	5.26	1.30×10^{-16}	9.20×10^{-15}	1.26×10^{-15}
**Glucose-Alanine Cycle**	13	2	65.87	5.26	6.78×10^{-12}	4.74×10^{-10}	5.87×10^{-11}
**Beta-Alanine Metabolism**	34	2	56.00	5.26	1.40×10^{-9}	9.64×10^{-8}	1.09×10^{-8}
**Glutathione Metabolism**	21	4	77.31	5.26	3.79×10^{-9}	2.58×10^{-7}	2.69×10^{-8}
**Propanoate Metabolism**	42	2	54.87	5.26	2.71×10^{-8}	1.81×10^{-6}	1.76×10^{-7}
**Selenoamino Acid Metabolism**	28	4	64.91	5.26	5.79×10^{-8}	3.82×10^{-6}	3.47×10^{-7}
**Carnitine Synthesis**	22	5	64.44	5.26	7.70×10^{-8}	5.01×10^{-6}	4.29×10^{-7}
**Lysine Degradation**	30	2	46.12	5.26	1.40×10^{-7}	8.95×10^{-6}	7.27×10^{-7}
**Urea Cycle**	29	6	61.14	5.26	1.52×10^{-7}	9.58×10^{-6}	7.41×10^{-7}
**Phospholipid Biosynthesis**	29	2	46.47	5.26	2.27×10^{-7}	1.41×10^{-5}	1.04×10^{-6}
**Tryptophan Metabolism**	60	4	60.76	5.26	3.82×10^{-7}	2.33×10^{-5}	1.66×10^{-6}
**Alanine Metabolism**	17	2	71.67	5.26	8.46×10^{-7}	5.08×10^{-5}	3.47×10^{-6}
**Purine Metabolism**	74	6	50.63	5.26	9.07×10^{-7}	5.35×10^{-5}	3.54×10^{-6}
**Aspartate Metabolism**	35	4	66.55	5.26	1.90×10^{-6}	1.10×10^{-4}	7.05×10^{-6}
**Betaine Metabolism**	21	5	56.74	5.26	1.99×10^{-6}	1.13×10^{-4}	7.05×10^{-6}
**Glutamate Metabolism**	49	6	57.97	5.26	3.67×10^{-6}	2.05×10^{-4}	1.24×10^{-5}
**Valine, Leucine and Isoleucine Degradation**	60	3	52.16	5.26	6.06×10^{-6}	3.33×10^{-4}	1.97×10^{-5}
**Thyroid hormone synthesis**	13	1	66.38	5.26	1.22×10^{-5}	6.58×10^{-4}	3.80×10^{-5}
**Arginine and Proline Metabolism**	53	7	51.62	5.26	1.28×10^{-5}	6.80×10^{-4}	3.85×10^{-5}
**Tyrosine Metabolism**	72	3	47.91	5.26	1.46×10^{-5}	7.60×10^{-4}	4.22×10^{-5}
**Glycine and Serine Metabolism**	59	10	54.75	5.26	1.67×10^{-5}	8.52×10^{-4}	4.66×10^{-5}
**Warburg Effect**	58	2	37.32	5.26	1.97×10^{-5}	9.83×10^{-4}	5.29×10^{-5}
**Phenylalanine and Tyrosine Metabolism**	28	2	63.26	5.26	2.48×10^{-5}	1.22×10^{-3}	6.16×10^{-5}
**Porphyrin Metabolism**	40	2	37.18	5.26	2.53×10^{-5}	1.22×10^{-3}	6.16×10^{-5}
**Bile Acid Biosynthesis**	65	2	37.18	5.26	2.53×10^{-5}	1.22×10^{-3}	6.16×10^{-5}
**Histidine Metabolism**	43	4	42.51	5.26	4.06×10^{-5}	1.87×10^{-3}	9.55×10^{-5}

**Biotin Metabolism**	8	1	61.59	5.26	4.18×10^{-5}	1.88×10^{-3}	9.55×10^{-5}
**Ammonia Recycling**	32	6	50.96	5.26	4.28×10^{-5}	1.88×10^{-3}	9.55×10^{-5}
**Methionine Metabolism**	43	10	51.75	5.26	4.45×10^{-5}	1.92×10^{-3}	9.65×10^{-5}
**Catecholamine Biosynthesis**	20	2	55.97	5.26	1.14×10^{-4}	4.78×10^{-3}	2.40×10^{-4}
**Sphingolipid Metabolism**	40	2	54.08	5.26	1.57×10^{-4}	6.42×10^{-3}	3.22×10^{-4}
**Estrone Metabolism**	24	2	35.17	5.26	1.67×10^{-4}	6.66×10^{-3}	3.33×10^{-4}
**Nicotinate and Nicotinamide Metabolism**	37	3	39.40	5.26	1.78×10^{-4}	6.96×10^{-3}	3.48×10^{-4}
**Homocysteine Degradation**	9	2	53.02	5.26	2.35×10^{-4}	8.95×10^{-3}	4.48×10^{-4}
**Phosphatidylcholine Biosynthesis**	14	2	52.77	5.26	2.55×10^{-4}	9.44×10^{-3}	4.74×10^{-4}
**Cysteine Metabolism**	26	2	33.31	5.26	2.71×10^{-4}	9.74×10^{-3}	4.91×10^{-4}
**Amino Sugar Metabolism**	33	1	48.14	5.26	6.91×10^{-4}	2.42×10^{-2}	1.20×10^{-3}
**Phenylacetate Metabolism**	9	1	48.14	5.26	6.91×10^{-4}	2.42×10^{-2}	1.20×10^{-3}
**Pyrimidine Metabolism**	59	2	33.03	5.26	9.66×10^{-4}	3.19×10^{-2}	1.64×10^{-3}
**Ubiquinone Biosynthesis**	20	1	42.93	5.26	1.72×10^{-3}	$5.49E \times 10^{-2}$	2.85×10^{-3}
**Spermidine and Spermine Biosynthesis**	18	5	38.59	5.26	1.77×10^{-3}	5.50×10^{-2}	2.88×10^{-3}
**Phosphatidylethanolamine Biosynthesis**	12	1	42.50	5.26	1.84×10^{-3}	5.53×10^{-2}	2.93×10^{-3}
**Pterine Biosynthesis**	29	1	25.63	5.26	2.27×10^{-2}	6.59×10^{-1}	2.30×10^{-2}
**Inositol Metabolism**	33	1	25.63	5.26	2.27×10^{-2}	6.59×10^{-1}	2.30×10^{-2}
**Ca eine Metabolism**	24	1	25.63	5.26	2.27×10^{-2}	6.59×10^{-1}	2.30×10^{-2}
**Glycerolipid Metabolism**	25	1	25.63	5.26	2.27×10^{-2}	6.59×10^{-1}	2.30×10^{-2}
**Fatty acid Metabolism**	43	1	25.63	5.26	2.27×10^{-2}	6.59×10^{-1}	2.30×10^{-2}
**Folate Metabolism**	29	1	25.63	5.26	2.27×10^{-2}	6.59×10^{-1}	2.30×10^{-2}
**Fatty Acid Elongation In Mictochondria**	35	1	25.63	5.26	2.27×10^{-2}	6.59×10^{-1}	2.30×10^{-2}
**Citric Acid Cycle**	32	1	25.63	5.26	2.27×10^{-2}	6.59×10^{-1}	2.30×10^{-2}
**Pyruvate Metabolism**	48	1	25.63	5.26	2.27×10^{-2}	6.59×10^{-1}	2.30×10^{-2}
**Androgen and Estrogen Metabolism**	33	1	25.63	5.26	2.27×10^{-2}	6.59×10^{-1}	2.30×10^{-2}
**Ketone Body Metabolism**	13	1	25.63	5.26	2.27×10^{-2}	6.59×10^{-1}	2.30×10^{-2}
**Butyrate Metabolism**	19	1	25.63	5.26	2.27×10^{-2}	6.59×10^{-1}	2.30×10^{-2}
**Glycerol Phosphate Shuttle**	11	1	25.63	5.26	2.27×10^{-2}	6.59×10^{-1}	2.30×10^{-2}
**Malate-Aspartate Shuttle**	10	1	25.63	5.26	2.27×10^{-2}	6.59×10^{-1}	2.30×10^{-2}
**Steroidogenesis**	43	1	25.63	5.26	2.27×10^{-2}	6.59×10^{-1}	2.30×10^{-2}
**Mitochondrial Electron Transport Chain**	19	1	25.63	5.26	2.27×10^{-2}	6.59×10^{-1}	2.30×10^{-2}
**Ethanol Degradation**	19	1	25.63	5.26	2.27×10^{-2}	6.59×10^{-1}	2.30×10^{-2}
**Phytanic Acid Peroxisomal Oxidation**	26	1	25.63	5.26	2.27×10^{-2}	6.59×10^{-1}	2.30×10^{-2}
**Threonine and 2-Oxobutonoate Degradation**	20	1	25.63	5.26	2.27×10^{-2}	6.59×10^{-1}	2.30×10^{-2}
**Inositol Phosphate Metabolism**	26	1	25.63	5.26	2.27×10^{-2}	6.59×10^{-1}	2.30×10^{-2}
**Transfer of Acetyl Groups into Mitochondria**	22	1	25.63	5.26	2.27×10^{-2}	6.59×10^{-1}	2.30×10^{-2}
**Plasmalogen Synthesis**	26	1	25.63	5.26	2.27×10^{-2}	6.59×10^{-1}	2.30×10^{-2}
**Mitochondrial Beta-Oxidation of Short Chain Saturated Fatty Acids**	27	1	25.63	5.26	2.27×10^{-2}	6.59×10^{-1}	2.30×10^{-2}
**Mitochondrial Beta-Oxidation of Medium Chain Saturated Fatty Acids**	27	1	25.63	5.26	2.27×10^{-2}	6.59×10^{-1}	2.30×10^{-2}
**Mitochondrial Beta-Oxidation of Long Chain Saturated Fatty Acids**	28	1	25.63	5.26	2.27×10^{-2}	6.59×10^{-1}	2.30×10^{-2}
**De Novo Triacylglycerol Biosynthesis**	9	1	25.63	5.26	2.27×10^{-2}	6.59×10^{-1}	2.30×10^{-2}
**Cardiolipin Biosynthesis**	11	1	25.63	5.26	2.27×10^{-2}	6.59×10^{-1}	2.30×10^{-2}
**Androstenedione Metabolism**	24	1	25.63	5.26	2.27×10^{-2}	6.59×10^{-1}	2.30×10^{-2}
**Oxidation of Branched Chain Fatty Acids**	26	1	14.94	5.26	9.22×10^{-2}	6.59×10^{-1}	9.22×10^{-2}

Materials and Methods

In vitro macrophage infections

All experiments were performed with 6–8-weeks-old female BALB/c mice obtained from the Animal Center of the Faculty of Medicine of the University of São Paulo and maintained in the Animal Center of Department of Physiology at the Institute of Bioscience of the University of São Paulo. The bone marrow-derived macrophages (BMDMs) were obtained from the femurs and tibias by flushing with 2 mL of PBS. Then, the cells were collected by centrifugation at 500× *g* for 10 min at 4 °C and resuspended in RPMI 1640 medium (LGC Biotecnologia, São Paulo, SP, Brazil), supplemented with penicillin (100 U/ml) (Invitrogen), streptomycin (100 μg/ml) (Invitrogen), 5% heat-inactivated FBS (Invitrogen) and 10% L929 cell supernatant. The cells were submitted to

differentiation for 7–8 days at 34 °C in an atmosphere of 5% CO_2. BMDMs were used after phenotypic analysis by flow cytometry (FACScalibur-Becton Dickinson, San Jose, CA, USA) demonstrated the presence of 95% F4/80- and CD11b-positive cells, confirming the macrophage differentiation.

The BMDMs were seeded into 8-well glass chamber slides (Lab-Teck Chamber Slide; Nunc, Naperville, IL, USA) (2×10^5/well) for infectivity analysis or into 6-well plates (SPL, Lifescience, Pocheon, Korea) (5×10^6/well) for metabolites analysis. After 18 h of incubation at 34 °C in an atmosphere of 5% CO_2, BMDMs were infected with La-WT or La-arg^- promastigotes in the stationary growth phase (MOI 5:1). After 4 h of infection, non-phagocyted promastigotes were washed with fresh medium and samples were collected for metabolites extraction or fixed for the infectivity indexes determination. The uninfected macrophages were maintained in the same conditions.

The infectivity was microscopically analyzed after cell-fixation with acetone/methanol (1:1, v:v, Merck, Darmstadt, Germany) for 20 min at −20 °C, followed by PBS washing and Panoptic-stained (Laborclin, Parana, Brazil). Infectivity was analyzed in phase-contrast microscopy (Nikon Eclipse E200, NJ, USA) counting the number of infected macrophages and amastigotes per macrophage in at least 1,000 macrophages/treatment in 3 independent experiments. The infection index was calculated by multiplying the mean number of amastigotes per macrophage by the rate of macrophage infection. The values were normalized based on the average values for the untreated infected macrophages.
